# 2,2,2-Tribromo-*N*-(2-chloro­phen­yl)acetamide

**DOI:** 10.1107/S1600536810001467

**Published:** 2010-01-16

**Authors:** B. Thimme Gowda, Sabine Foro, P. A. Suchetan, Hartmut Fuess

**Affiliations:** a Department of Chemistry, Mangalore University, Mangalagangotri 574 199, Mangalore, India; b Institute of Materials Science, Darmstadt University of Technology, Petersenstrasse 23, D-64287 Darmstadt, Germany

## Abstract

In the title compound, C_8_H_5_Br_3_ClNO, the conformation of the N—H bond is *syn* to the 2-chloro substituent in the benzene ring. There are no classical inter­molecular hydrogen bonds, but intra­molecular N—H⋯Br and N—H⋯Cl contacts occur.

## Related literature

For preparation of the title compound, see: Gowda *et al.* (2003 ▶). For background to our studies on the effect of the ring and the side-chain substituents on the crystal structures of *N*-aromatic amides, see: Gowda *et al.* (2007 ▶, 2009 ▶). For the conformations of other amides, see: Brown (1966 ▶).
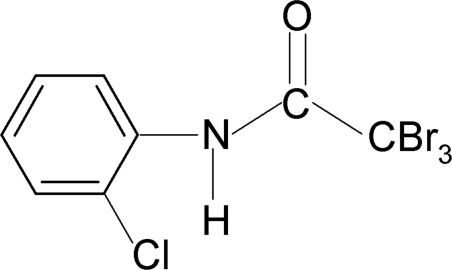


## Experimental

### 

#### Crystal data

C_8_H_5_Br_3_ClNO
*M* *_r_* = 406.31
Orthorhombic, 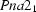

*a* = 9.1947 (6) Å
*b* = 12.9645 (7) Å
*c* = 9.5213 (6) Å
*V* = 1134.98 (12) Å^3^
*Z* = 4
Mo *K*α radiation
μ = 10.86 mm^−1^
*T* = 299 K
0.40 × 0.40 × 0.34 mm

#### Data collection

Oxford Diffraction Xcalibur diffractometer with a Sapphire CCD detector
Absorption correction: multi-scan (*CrysAlis RED*; Oxford Diffraction, 2009 ▶) *T*_min_ = 0.098, *T*_max_ = 0.120
4524 measured reflections
1645 independent reflections
1491 reflections with *I* > 2σ(*I*)
*R*_int_ = 0.022

#### Refinement

*R*[*F* ^2^ > 2σ(*F* ^2^)] = 0.027
*wR*(*F* ^2^) = 0.066
*S* = 1.07
1645 reflections
131 parameters
2 restraints
H atoms treated by a mixture of independent and constrained refinement
Δρ_max_ = 0.77 e Å^−3^
Δρ_min_ = −0.56 e Å^−3^
Absolute structure: Flack (1983 ▶), 416 Friedel pairs
Flack parameter: 0.049 (18)

### 

Data collection: *CrysAlis CCD* (Oxford Diffraction, 2009 ▶); cell refinement: *CrysAlis RED* (Oxford Diffraction, 2009 ▶); data reduction: *CrysAlis RED*; program(s) used to solve structure: *SHELXS97* (Sheldrick, 2008 ▶); program(s) used to refine structure: *SHELXL97* (Sheldrick, 2008 ▶); molecular graphics: *PLATON* (Spek, 2009 ▶); software used to prepare material for publication: *SHELXL97*.

## Supplementary Material

Crystal structure: contains datablocks I, global. DOI: 10.1107/S1600536810001467/bt5167sup1.cif

Structure factors: contains datablocks I. DOI: 10.1107/S1600536810001467/bt5167Isup2.hkl

Additional supplementary materials:  crystallographic information; 3D view; checkCIF report

## Figures and Tables

**Table 1:** Hydrogen-bond geometry (Å, °)

*D*—H⋯*A*	*D*—H	H⋯*A*	*D*⋯*A*	*D*—H⋯*A*
N1—H1*N*⋯Br1	0.85 (3)	2.78 (8)	3.155 (6)	109 (6)
N1—H1*N*⋯Cl1	0.85 (3)	2.59 (7)	2.961 (5)	107 (5)

**Fig. 1.**
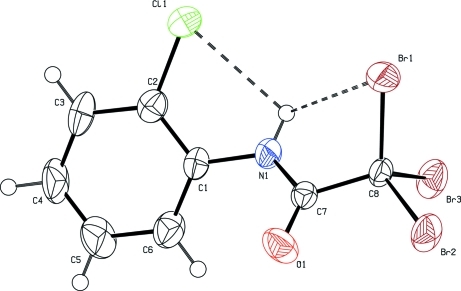
Molecular structure of the title compound, showing the atom labelling scheme. Displacement ellipsoids are drawn at the 50% probability level and the H atoms are represented as small spheres of arbitrary radii.

**Fig. 2.**
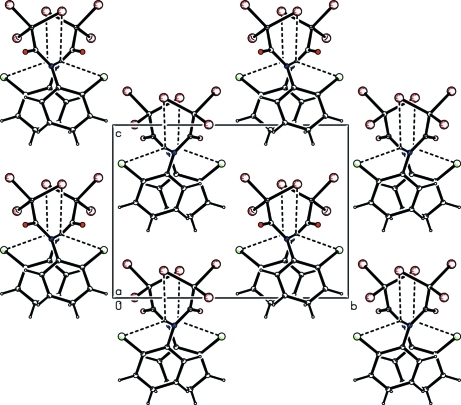
Molecular packing of the title compound with hydrogen bonds shown as dashed lines.

## supplementary crystallographic information

### Comment

As a part of studying the effect of the ring and the side chain substituents on the crystal structures of *N*-aromatic amides (Gowda *et al.*, 2007, 2009), in the present work, the structure of *N*-(2-chlorophenyl)2,2,2-tribromoacetamide (I) has been determined (Fig.1). The conformation of the N—H bond is *syn* to the 2-chloro substituent in the benzene ring, similar to that observed in *N*-(2-chlorophenyl)acetamide and *N*-(2-chlorophenyl)2,2,2-trichloroacetamide (Gowda *et al.*, 2007), but contrary to the *anti* conformation observed between the N—H bond and the 3-methyl group in *N*-(3-methylphenyl)2,2,2-tribromoacetamide (Gowda *et al.*, 2009). Further, the conformation of the N—H bond in the structure is *anti* to the C=O bond in the side chain, similar to that observed in *N*-(phenyl)2,2,2-tribromoacetamide (Gowda *et al.*, 2009) and other amides (Brown, 1966; Gowda *et al.*, 2007, 2009). The structure   shows simultaneous N—H···Br and N—H···Cl intramolecular H-bonding. The packing diagram of the molecules is shown in Fig. 2.

### Experimental

The title compound was prepared from 2-chloroaniline, tribromoacetic acid and phosphorylchloride according to the literature method (Gowda *et al.*, 2003). The purity of the compound was checked by determining its melting point. It was further characterized by recording its infrared spectra. Single crystals of the title compound used for X-ray diffraction studies were obtained by a slow evaporation of its ethanolic solution at room temperature.

### Refinement

The H atom of the NH group was located in a difference map and later restrained to the distance N—H = 0.86 (3) Å. The other H atoms were positioned with idealized geometry using a riding model [C—H = 0.93 Å]. All H atoms were refined with isotropic displacement parameters  set to 1.2 times of the *U*_eq_ of the parent atom.

### Crystal data

**Table:**

C_8_H_5_Br_3_ClNO	*F*(000) = 760
*M**_r_* = 406.31	*D*_x_ = 2.378 Mg m^−^^3^
Orthorhombic, *P**n**a*2_1_	Mo *K*α radiation, λ = 0.71073 Å
Hall symbol: P 2c -2n	Cell parameters from 3507 reflections
*a* = 9.1947 (6) Å	θ = 2.7–27.8°
*b* = 12.9645 (7) Å	µ = 10.86 mm^−^^1^
*c* = 9.5213 (6) Å	*T* = 299 K
*V* = 1134.98 (12) Å^3^	Rod, colourless
*Z* = 4	0.40 × 0.40 × 0.34 mm

### Data collection

**Table:**

Oxford Diffraction Xcalibur diffractometer with a Sapphire CCD detector	1645 independent reflections
Radiation source: fine-focus sealed tube	1491 reflections with *I* > 2σ(*I*)
graphite	*R*_int_ = 0.022
Rotation method data acquisition using ω and φ scans.	θ_max_ = 26.4°, θ_min_ = 2.7°
Absorption correction: multi-scan (*CrysAlis RED*; Oxford Diffraction, 2009)	*h* = −11→11
*T*_min_ = 0.098, *T*_max_ = 0.120	*k* = −16→15
4524 measured reflections	*l* = −11→6

### Refinement

**Table:**

Refinement on *F*^2^	Hydrogen site location: inferred from neighbouring sites
Least-squares matrix: full	H atoms treated by a mixture of independent and constrained refinement
*R*[*F*^2^ > 2σ(*F*^2^)] = 0.027	*w* = 1/[σ^2^(*F*_o_^2^) + (0.0357*P*)^2^ + 1.2795*P*] where *P* = (*F*_o_^2^ + 2*F*_c_^2^)/3
*wR*(*F*^2^) = 0.066	(Δ/σ)_max_ = 0.004
*S* = 1.07	Δρ_max_ = 0.77 e Å^−^^3^
1645 reflections	Δρ_min_ = −0.56 e Å^−^^3^
131 parameters	Extinction correction: *SHELXL97* (Sheldrick, 2008), Fc^*^=kFc[1+0.001xFc^2^λ^3^/sin(2θ)]^-1/4^
2 restraints	Extinction coefficient: 0.0072 (5)
Primary atom site location: structure-invariant direct methods	Absolute structure: Flack (1983), 416 Friedel pairs
Secondary atom site location: difference Fourier map	Flack parameter: 0.049 (18)

### Special details

**Table:**

Experimental. CrysAlis RED (Oxford Diffraction, 2009) Empirical absorption correction using spherical harmonics, implemented in SCALE3 ABSPACK scaling algorithm.
Geometry. All e.s.d.'s (except the e.s.d. in the dihedral angle between two l.s. planes) are estimated using the full covariance matrix. The cell e.s.d.'s are taken into account individually in the estimation of e.s.d.'s in distances, angles and torsion angles; correlations between e.s.d.'s in cell parameters are only used when they are defined by crystal symmetry. An approximate (isotropic) treatment of cell e.s.d.'s is used for estimating e.s.d.'s involving l.s. planes.
Refinement. Refinement of *F*^2^ against ALL reflections. The weighted *R*-factor *wR* and goodness of fit *S* are based on *F*^2^, conventional *R*-factors *R* are based on *F*, with *F* set to zero for negative *F*^2^. The threshold expression of *F*^2^ > σ(*F*^2^) is used only for calculating *R*-factors(gt) *etc*. and is not relevant to the choice of reflections for refinement. *R*-factors based on *F*^2^ are statistically about twice as large as those based on *F*, and *R*- factors based on ALL data will be even larger.

### Fractional atomic coordinates and isotropic or equivalent isotropic displacement parameters (Å^2^)

**Table:**

	*x*	*y*	*z*	*U*_iso_*/*U*_eq_	
Br1	0.56049 (8)	0.28366 (5)	1.15778 (8)	0.0510 (2)	
Br2	0.43394 (7)	0.06152 (5)	1.19542 (7)	0.0483 (2)	
Br3	0.70743 (6)	0.09279 (5)	1.00260 (9)	0.0474 (2)	
Cl1	0.5017 (2)	0.46215 (12)	0.7770 (2)	0.0536 (5)	
O1	0.3140 (5)	0.1287 (4)	0.9233 (5)	0.0521 (13)	
N1	0.4832 (5)	0.2390 (4)	0.8407 (6)	0.0366 (12)	
H1N	0.554 (5)	0.277 (4)	0.866 (9)	0.044*	
C1	0.4101 (6)	0.2689 (4)	0.7164 (7)	0.0311 (12)	
C2	0.4114 (6)	0.3709 (4)	0.6763 (7)	0.0360 (14)	
C3	0.3435 (8)	0.4026 (5)	0.5526 (7)	0.0475 (17)	
H3	0.3476	0.4712	0.5244	0.057*	
C4	0.2701 (8)	0.3305 (6)	0.4724 (8)	0.0573 (19)	
H4	0.2235	0.3506	0.3901	0.069*	
C5	0.2660 (9)	0.2308 (6)	0.5136 (9)	0.063 (2)	
H5	0.2152	0.1829	0.4598	0.076*	
C6	0.3362 (8)	0.1987 (5)	0.6347 (7)	0.0471 (17)	
H6	0.3335	0.1296	0.6607	0.057*	
C7	0.4282 (6)	0.1716 (4)	0.9346 (6)	0.0278 (12)	
C8	0.5253 (6)	0.1542 (4)	1.0638 (7)	0.0288 (12)	

### Atomic displacement parameters (Å^2^)

**Table:**

	*U*^11^	*U*^22^	*U*^33^	*U*^12^	*U*^13^	*U*^23^
Br1	0.0744 (5)	0.0373 (3)	0.0411 (4)	−0.0059 (3)	−0.0169 (4)	−0.0036 (3)
Br2	0.0476 (4)	0.0509 (4)	0.0464 (4)	−0.0058 (3)	0.0000 (3)	0.0248 (3)
Br3	0.0328 (3)	0.0471 (4)	0.0623 (5)	0.0097 (3)	0.0018 (3)	0.0139 (4)
Cl1	0.0620 (10)	0.0384 (8)	0.0604 (12)	−0.0142 (8)	−0.0155 (9)	0.0153 (8)
O1	0.047 (3)	0.067 (3)	0.043 (3)	−0.025 (2)	−0.009 (2)	0.011 (3)
N1	0.040 (3)	0.035 (3)	0.035 (3)	−0.007 (2)	−0.008 (2)	0.013 (3)
C1	0.035 (3)	0.032 (3)	0.026 (3)	0.008 (2)	0.000 (2)	0.004 (2)
C2	0.032 (3)	0.036 (3)	0.040 (4)	0.004 (2)	0.004 (3)	0.004 (3)
C3	0.057 (4)	0.052 (4)	0.033 (4)	0.018 (3)	0.004 (3)	0.018 (3)
C4	0.074 (5)	0.070 (5)	0.028 (4)	0.010 (4)	−0.017 (4)	0.012 (4)
C5	0.092 (5)	0.065 (5)	0.033 (4)	0.011 (4)	−0.020 (4)	−0.016 (4)
C6	0.068 (4)	0.039 (3)	0.034 (4)	0.011 (3)	−0.005 (4)	−0.002 (3)
C7	0.028 (3)	0.027 (3)	0.029 (3)	0.002 (2)	0.000 (2)	0.000 (2)
C8	0.032 (3)	0.027 (3)	0.027 (3)	0.001 (2)	−0.001 (2)	0.008 (2)

### Geometric parameters (Å, °)

**Table:**

Br1—C8	1.929 (6)	C2—C3	1.395 (9)
Br2—C8	1.929 (6)	C3—C4	1.383 (10)
Br3—C8	1.944 (6)	C3—H3	0.9300
Cl1—C2	1.735 (7)	C4—C5	1.351 (11)
O1—C7	1.193 (6)	C4—H4	0.9300
N1—C7	1.349 (7)	C5—C6	1.385 (10)
N1—C1	1.415 (8)	C5—H5	0.9300
N1—H1N	0.85 (3)	C6—H6	0.9300
C1—C6	1.377 (9)	C7—C8	1.537 (8)
C1—C2	1.376 (8)		
			
C7—N1—C1	123.6 (5)	C4—C5—C6	121.1 (7)
C7—N1—H1N	118 (6)	C4—C5—H5	119.5
C1—N1—H1N	116 (5)	C6—C5—H5	119.5
C6—C1—C2	118.8 (6)	C1—C6—C5	120.1 (6)
C6—C1—N1	121.7 (5)	C1—C6—H6	119.9
C2—C1—N1	119.4 (6)	C5—C6—H6	119.9
C1—C2—C3	120.9 (6)	O1—C7—N1	124.9 (6)
C1—C2—Cl1	120.4 (5)	O1—C7—C8	121.0 (5)
C3—C2—Cl1	118.7 (5)	N1—C7—C8	114.1 (4)
C4—C3—C2	119.0 (6)	C7—C8—Br1	110.0 (4)
C4—C3—H3	120.5	C7—C8—Br2	111.0 (4)
C2—C3—H3	120.5	Br1—C8—Br2	108.3 (3)
C5—C4—C3	120.0 (6)	C7—C8—Br3	108.7 (4)
C5—C4—H4	120.0	Br1—C8—Br3	110.5 (3)
C3—C4—H4	120.0	Br2—C8—Br3	108.3 (3)
			
C7—N1—C1—C6	−41.6 (9)	N1—C1—C6—C5	−179.9 (6)
C7—N1—C1—C2	137.9 (6)	C4—C5—C6—C1	0.9 (12)
C6—C1—C2—C3	−2.1 (9)	C1—N1—C7—O1	1.1 (9)
N1—C1—C2—C3	178.4 (6)	C1—N1—C7—C8	−177.9 (5)
C6—C1—C2—Cl1	179.4 (5)	O1—C7—C8—Br1	−121.3 (5)
N1—C1—C2—Cl1	−0.1 (8)	N1—C7—C8—Br1	57.7 (6)
C1—C2—C3—C4	2.2 (10)	O1—C7—C8—Br2	−1.5 (7)
Cl1—C2—C3—C4	−179.4 (5)	N1—C7—C8—Br2	177.5 (4)
C2—C3—C4—C5	−0.7 (11)	O1—C7—C8—Br3	117.5 (5)
C3—C4—C5—C6	−0.9 (12)	N1—C7—C8—Br3	−63.4 (5)
C2—C1—C6—C5	0.6 (10)		

### Hydrogen-bond geometry (Å, °)

**Table:**

*D*—H···*A*	*D*—H	H···*A*	*D*···*A*	*D*—H···*A*
N1—H1N···Br1	0.85 (3)	2.78 (8)	3.155 (6)	109 (6)
N1—H1N···Cl1	0.85 (3)	2.59 (7)	2.961 (5)	107 (5)
